# Electro-Conductive Composite Gold-Polyethersulfone-Ultrafiltration-Membrane: Characterization of Membrane and Natural Organic Matter (NOM) Filtration Performance at Different In-Situ Applied Surface Potentials

**DOI:** 10.3390/membranes8030064

**Published:** 2018-08-16

**Authors:** Tomi Mantel, Paul Benne, Stanislav Parsin, Mathias Ernst

**Affiliations:** Institute for Water Resources and Water Supply, Hamburg University of Technology, Am Schwarzenberg-Campus 3, 20173 Hamburg, Germany; paul.benne@tuhh.de (P.B.); stanislav.parsin@tuhh.de (S.P.); mathias.ernst@tuhh.de (M.E.)

**Keywords:** electro-conductive membrane, electro-enhanced rejection, surface charge, membrane characterization, electro-ultrafiltration, surface coating

## Abstract

Next to the pore size distribution, surface charge is considered to be one main factor in the separation performance of ultrafiltration (UF) membranes. By applying an external surface potential onto an electro-conductive UF membrane, electrostatic induced rejection was investigated. This study introduces in a first part a relatively simple but yet not reported technology of membrane modification with direct current sputter deposition of ultrathin (15 nm) highly conductive gold layers. In a second part, characterization of the gold-coated UF flat sheet membrane with a molecular weight cut-off (MWCO) of 150 kDa is presented. Membrane parameters as contact angle (hydrophobicity), pure water permeability, MWCO, scanning electron microscopy imaging, zeta potential, surface conductivity and cyclic voltammetry of the virgin and the modified membrane are compared. Due to the coating, a high surface conductivity of 10^7^ S m^−1^ was realized. Permeability of the modified membrane decreased by 40% but MWCO and contact angle remained almost unchanged. In a third part, cross-flow filtration experiments with negative charged Suwannee River Natural Organic Matter (SRNOM) are conducted at different cathodic and anodic applied potentials, different pH values (pH 4, 7, 10) and ionic strengths (0, 1, 10 mmol L^−1^). SRNOM rejection of not externally charged membrane was 28% in cross-flow and 5% in dead-end mode. Externally negative charged membrane (−1.5 V vs. Ag/AgCl) reached rejection of 64% which was close to the performance of commercial UF membrane with MWCO of 5 kDa. High ionic strengths or low pH of feed reduced the effect of electrostatic rejection.

## 1. Introduction

Ultrafiltration (UF) is an established technology for water treatment due to its high permeability at sufficient rejection rates for turbidity and pathogens as well as its relatively compact footprint [1,2]. Especially if it comes to tight UF, rejection mechanisms are not entirely understood, because rejection rates cannot simply be explained by size exclusion effects [3]. Next to the nominal pore size and pore size distribution of a UF membrane, surface charge is considered to be a relevant factor in influencing separation performance of UF membranes [4]. Several studies have been published about the influence of the membranes surface zeta potential on fouling and rejection behavior of UF membranes. Accordingly, negative membrane charge (identified by measured negative zeta potential) is considered to result in higher rejection rates for negatively charged substances such as natural organic matter (NOM). Moreover, lower fouling rates have been reported under such surface charge conditions [5,6]. 

By the utilization of an electrically conductive UF membrane, external in-situ charging of membrane becomes possible during filtration without changing of any other membrane properties. Using this novel approach, the impact of membrane surface potential on filtration performance can be evaluated for different applied cathodic and anodic potentials; furthermore, the membrane surface potential can be changed during a running experiment which leads to new experimental opportunities to study the impact of surface charge.

In the past five years, research on electrically conductive membranes (ECM) increased considerably. ECMs have been proposed and investigated for rejection enhancement as well as fouling control. Several research groups showed the application of novel developed ECMs for water treatment in the context of fouling mitigation and scaling inhibition [7,8,9]. 

For this study, an electro-conductive UF membrane was prepared by sputter coating of a commercial polymer UF flat sheet membrane. Sputter deposition is a widespread and economic method to create ultrathin conducting layers for scientific (e.g., scanning electron microscopy (SEM) imaging) or industrial (e.g., thin films) application [10]. DC sputtering of gold proved to be a simple and consistent method to create an ultrathin conducting layer on the membrane surface. Sputter coating of membranes with different metals as palladium or platinum were reported by a few studies; however, such membranes were used for hydrogen gas separation [11] or the coating was primarily for analytical purpose (e.g., in-situ analyses of fouling of UF membrane by measuring double layer potential [12,13]). Accordingly, no external electrostatic potential was applied to conductive metal-polymer-composite membranes during water filtration experiments so far.

The present study focuses on the investigation of the electrostatically induced rejection mechanisms by the external control of cathodic and anodic surface potentials; the filtration experiments are conducted with negative charged NOMs as model foulants, which are typically found in natural water resources. Moreover, impact of water matrix (ionic strength) and pH are investigated with respect to the electrostatically enhanced rejection mechanism. By this, a possible functionalization of the membrane performance with external in-situ charging is targeted.

## 2. Materials and Methods 

### 2.1. Preparation of Gold-Polymer-Composite Membrane

Polyethersulfone (PES) UF flat sheet membrane UP150 with a molecular weight cutoff (MWCO) of 150 kDa and nominal pore size of 26 nm was applied (Microdyn-Nadir GmbH, Wiesbaden, Germany). The membrane was cut into rectangular sheets (100 mm × 50 mm) and then coated without any previous treatment with a direct current sputter coater on the active layer with gold (Sputter Coater SCD 005, Baltec Inc., Balzers, Lichtenstein). Sputter deposition was carried out in intervals of 5 nm gold layer thickness and then stopped for 10 s. This procedure was repeated for three times to accomplish a final gold layer thickness of 15 nm. The duration for the complete coating of 15 nm gold was approximately 180 seconds. The growth rate of the gold layer on the membrane surface was determined with a quartz micro balance (EM QSG100, Leica, Germany) and was approximately 0.1 nm s^−1^. DC sputter current was set at 40 mA, sputter voltage was 400–500 V, working pressure was 5 Pa, working distance was 50 mm and the operating gas was argon. After the coating process, the membranes were rinsed in purified water (Milli-Q, Millipore Corporation, Billerica, MA, USA) for at least 24 h. Prior to experiments, membranes were washed by filtration of at least 1 L of pure water.

### 2.2. Membrane Characterization

#### 2.2.1. Pure Water Permeability

Pure water permeability was measured for each membrane before every experiment at a transmembrane pressure (TMP) of 1 bar and a cross-flow velocity of 0.16 m s^−1^ (three virgin membranes and three Au-coated membranes with 5, 10 and 15 nm). Calculation followed Equation (1) with $\dot{V}$ as permeate volume flow, *A* as membrane area and *p* as pressure.

(1) $$P=\frac{\dot{V}}{A\;p\;t}\;\left[\frac{L}{m^{2}bar\;h}\right]$$

#### 2.2.2. Hydrophobicity—Contact Angle

Contact angle was measured by captive bubble method [14] with a self-assembled microscope setup. Experiments were conducted 12 times (or more) with random pieces from virgin membranes and 15 nm gold coated membranes. The dimensions of the membrane pieces were 20 mm × 30 mm. For graphical evaluation of the contact angle, the software Surftens (OEG GmbH, Frankfurt an der Oder, Germany) was used. Additional information is supplied in supplementary material.

#### 2.2.3. Scanning Electron Microscopy

Scanning electron microscopy (SEM) imaging was conducted at the electron microscopy unit (BEEM) of Hamburg University of Technology with Zeiss Leo Gemini 1530 (Carl Zeiss AG, Oberkochen, Germany). Before microscopy, virgin membranes where coated with 1.5 nm of gold to create contrast for SEM imaging (Sputter Coater SCD 005). Conductive membranes were not coated any further. The acceleration voltage was 2.0 kV.

#### 2.2.4. Zeta Potential

Zeta potential of virgin and coated membrane was investigated with Surpass (Anton Paar GmbH, Graz, Austria) at 1 mol L^−1^ KCl. Measurement started at pH 9 and through titration of HCl, the pH was decreased until pH 3. Membranes were cut into the dimensions of gap cell (20 mm × 10 mm) and stored in pure water for 24 h before the measurement. Zeta potential was calculated according to the streaming potential method by Helmholtz–Smoluchowski equation with the Software Attract supplied by the manufacturer of Surpass [15]. For more detailed information, the following reference is recommended [16]. 

#### 2.2.5. Molecular Weight Cut-Off 

For MWCO determination of the coated and uncoated membrane, polyethylene glycol (PEG) standards were used with molecular weights of 12.3, 26.1, 42.7, 98 and 200 g mol^−1^ (or kDa) and one dextran standard with 130 kDa (PSS Polymer Standards Service GmbH, Mainz, Germany). Rejection is calculated according to Equation (2). In order to avoid concentration polarization effects, permeate samples were taken after 20 mL of filtration. Experiments were done in stirred dead-end cells (Amicon Model 8200, Millipore Corp., Billerica, MA, USA) with a volume of 200 mL, active membrane surface area of 28.7 cm^2^, TMP of 1 bar and stirring speed of 300 rpm. Concentration of feed solution was set to 20 mg L^−1^. Measurement of PEGs and dextran concentration was conducted via the total organic carbon (TOC) detection of TOC-Analyzer (TOC Shimadzu, TOC-L CPH, Kyoto, Japan). Prior to the MWCO experiments, 1 L of pure water was filtered through the membrane to clean them.

(2) $$R=\left(1-\frac{c_{Permeate}}{c_{Feed}}\right)\cdot 100\%$$

#### 2.2.6. Electrical Properties of Conductive Membrane Electrode

The electrical conductivity of the membrane was measured following the Van der Pauw method (four-point method) [17]. At least three random membrane pieces of each membrane (respectively gold layer thickness 5, 10 and 15 nm) were chosen and measured for at least ten times.

Cyclic voltammetry (CV) was carried out using a three-electrode system with Ag/AgCl electrode (Xylem Analytics GmbH, Meinsberg, Germany) as reference electrode and coated active layer of membrane as working electrode and stainless steel sheet as counter electrode. The electrode area was 42 cm^2^. For the setup of parameters for cyclic voltammetry, EcmWin-Software (IPS Elektroniklabor GmbH, Münster, Germany) was used. The CV scan rate was 100 mV s^−1^ over a potential range of –4.0 to +4.0 V vs. Ag/AgCl. Measurements were conducted with Suwannee River Natural Organic Matter (SRNOM) solution (see Section 2.3.2) at sodium chloride concentrations of 0.1, 1 and 10 mmol L^−1^. The scan started at 0 V in the positive direction and was executed for 3 cycles.

### 2.3. Experimental Setup and Filtration Experiments

#### 2.3.1. Filtration Setup

**Figure 1:**
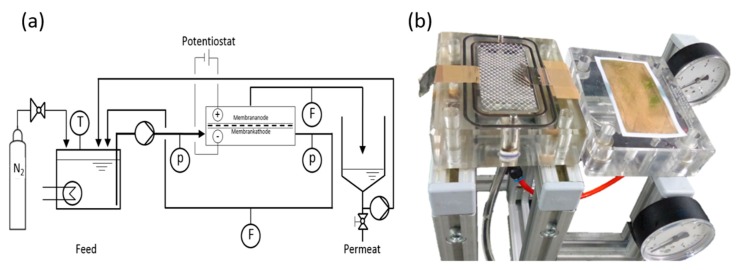
(**a**) Filtration setup; (**b**) Opened cross-flow cell (modified CF042) with titanium contacts, counter electrode, spacer and gold coated ultrafiltration (UF) membrane.

Filtration experiments were carried out using a commercially available acrylic flat-sheet cross-flow cell (CF042 Sterlitech, Kent, WA, USA) with an active membrane surface area of 42 cm^2^ (Figure 1). For contacting the electrodes, titanium foils are introduced into the cell and are connected to the potentiostat (PGU, IPS Elektroniklabor GmbH, Münster, Germany).

For dead-end mode, the cross-flow pump (MCP-Z Ismatech, Wertheim, Germany) is not operated and the pressure is applied through nitrogen gas and a pressure control valve (Swagelok, OH, USA). For cross-flow experiments, the cross-flow pump operates at a volume flow of 400 mL min^−1^, which leads to a cross-flow velocity of approximately 0.16 m s^−1^. The permeate is pumped back to the feed and pressure tank of 20 L volume (Millipore Corp., Billerica, MA, USA). In all experiments, a constant transmembrane pressure (TMP) of 1 bar was applied and permeate flow was controlled by a variable area flowmeter (Kobold Messring GmbH, Hofheim, Germany). The temperature of the feed solution was maintained under constant ambient conditions (20–22 °C). As counter electrode, a stainless steel sheet of 0.5 mm thickness is used. To avoid shortcuts between membrane-electrode and counter electrode, a plastic mesh spacer with a thickness of 1 mm is placed between both electrodes. The distance between the electrodes is 2 mm.

#### 2.3.2. Feed Solution 

SRNOM Standards (2R101N) was obtained from the International Humic Substances Society (IHSS, Denver, CO, USA). The SRNOM is a well-described NOM standard with a known size distribution from 0.3–500 g mol^−1^ including low molecular fulvic acids and high molecular humic acids [18,19]. Because of the wide molecular weight distribution, SRNOM is suitable to characterize the rejection performance of a UF membrane with UV-VIS method [18]. The stock solution was prepared at 100 mg L^−1^ in pure water and dissolved overnight at room temperature with stirring at 300 rpm and then filtered by syringe through a 0.20 μm membrane (DuraPES200, Membrana GmbH, Wuppertal, Germany). Feed solution was prepared by diluting the stock solution to a concentration of 6 mg L^−1^. The pH was adjusted with sodium hydroxide or hydrochloric acid (Carl Roth GmbH + Co KG, Karlsruhe, Germany) and ionic strength of the solution was elevated by adding sodium chloride (Carl Roth GmbH + Co KG, Germany). Electric conductivity and pH were determined with LF 315 and pH 340i (WTW Xylem GmbH, Weilheim, Germany), respectively.

#### 2.3.3. Filtration Experiments

The impact of pH, the ionic strength of feed solution as well as the applied membrane surface potential on filtration performance was investigated. All experiments are conducted as triplicates (or more). Surface potential of the electrode was measured by an additional experiment with Ag/AgCl reference electrode (Xylem Analytics GmbH, Meinsberg, Germany) in a beaker, as the electrode could not be directly placed into the filtration cell (see Section 2.2.6). It was tested neutral, negative and positive surface potentials for filtration experiments (Table 1). For this, the membrane was used as a flow-through electrode (either cathode or anode). The samples from cross-flow filtration experiments were taken after 250 mL of permeate volume. Rejection of NOM was measured through TOC (Shimadzu, TOC-L CPH) and spectral absorption coefficient at a wavelength of 254 nm (UV_254_ or SAC_254_) with a 50 mm cuvette (DR500, Hach Lange, Düsseldorf, Germany). UV_254_ is proved to have good linear correlation with TOC of SRNOM [3,7,20] and was primarily used for rejection calculation. Filtration volume is chosen small to avoid the influence of NOM fouling on membrane rejection by adsorption onto the membrane surface and into the pores [5,21]. In other experiments, the membrane was first charged negatively and after 250 mL of filtration, charge was switched positively (e.g., from −1 to +1 V vs. Ag/AgCl). Immediately after the change of electrical polarity, samples were taken.

Prior to experiments, the membranes were rinsed in pure water (Milli-Q) for at least 24 h. Membranes that were used in a previous experiment were cleaned in sodium hydroxide (pH 11.5) solution for 24 h and used again if the initial pure water permeability was reached. Investigated parameters of filtration experiments are shown in Table 1. All experiments were conducted at TMP of 1 bar and a cross-flow of 400 mL min^−1^, equivalent to a cross-flow velocity of 0.16 m s^−1^.

In order to evaluate the SRNOM rejection of the externally charged gold-UP150, additional cross-flow filtration experiments—with the same filtration parameters but with tighter UF membranes—were conducted (PES-UP020, MWCO 20 kDa, pore size 11 nm and PES-UP005, MWCO 5 kDa, pore size 6 nm, both Microdyn-Nadir, Wiesbaden, Germany). 

## 3. Results

### 3.1. Membrane Characterization

**Figure 2:**
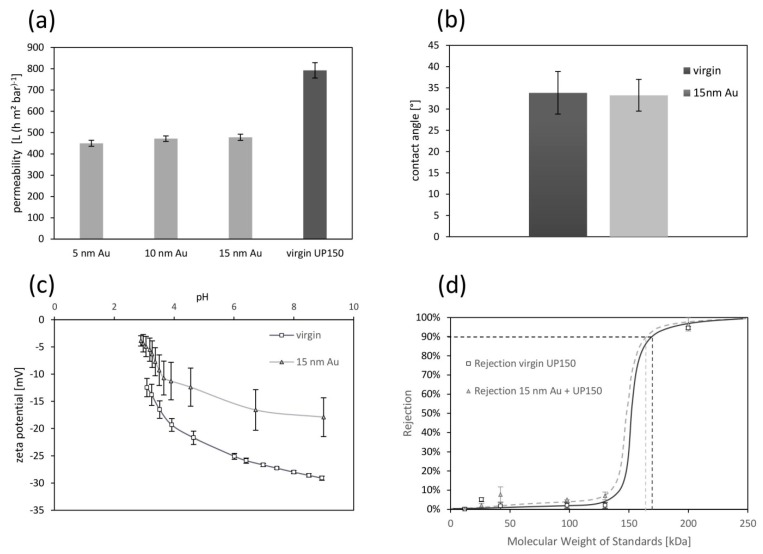
Membrane characterization virgin vs. gold coated UP150: (**a**) pure water permeability; (**b**) contact angle; (**c**) Zeta potential at 1 mol L^−1^ KCl; (**d**) molecular weight cut-off with estimated separation curves, error bars present standard deviations.

Due to the sputter coating of the membrane, pure water permeability decreases by 40% compared to the virgin membrane (Figure 2a). Similar results were obtained by Gaedt et al. [12] after membrane coating. However, the thickness of the gold layer (5, 10 or 15 nm) obviously did not affect the resulting permeability considerably. The coating of the membrane (Figure 2b) does not visibly change the contact angle. As expected, the virgin PES membrane showed a negative surface charge. By coating with gold, the zeta potential increased (less negative) compared to the virgin membrane (Figure 2c). From the error bars, it can be seen that Au-surface coating led to considerably higher measuring inaccuracy of zeta potential. Likely, the decreased roughness of the membrane surface is a reason for this [16]. Literature data of the zeta potential of gold surfaces [22] shows similar zeta potential properties as the sputtered membrane. Differences in MWCO of the coated membrane and virgin membrane are rather small or not existing, following the definition, that 90% rejection marks the nominal MWCO (Figure 2d).

**Figure 3:**
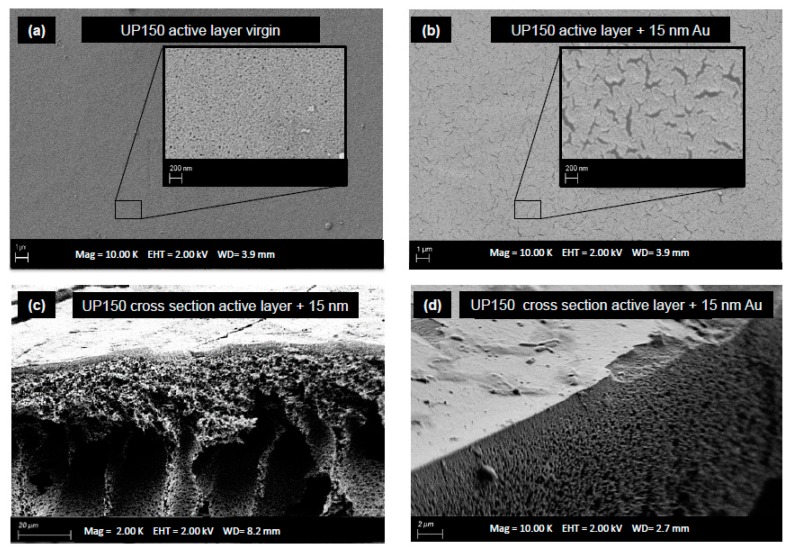
Scanning electron microscopy (SEM) imaging: (**a**) virgin UP150 with 3.6 nm Au coating for SEM contrast, 10 k× and 100 k×; (**b**) UP150 + 15 nm Au, 10 k× and 100 k×; (**c**) cross section of coated membrane, 2 k×; (**d**) cross section of active layer + 15 nm Au,10 k×.

SEM imaging gives an impression of the pore size and the pore size distribution of the virgin membrane (Figure 3a) and the coated membrane (Figure 3b). No annular pores can be identified after the surface modification by a 15 nm gold layer. The Au-surface seems to be generally porous and cracked. Size of pores of the virgin UP150 is given by the manufacturer at 26 nm. This was confirmed by the SEM imaging. The lengths of cracks in the gold layer are about 1–2 µm and width is approximately 0.05 µm. However, for the SEM imaging, the membrane had to be dried first which could have led to an enlargement of the cracks due to the shrinking of the membrane.

**Figure 4:**
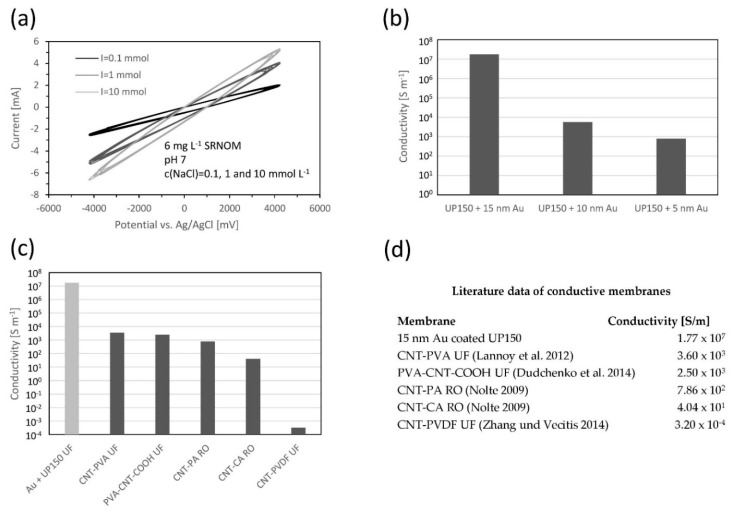
Electrochemical characterization of membrane: (**a**) cyclic voltammetry of Au coated UP150 in Suwannee River Natural Organic Matter (SRNOM) solution of 6 mg L^−1^ at 0.1, 1 and 10 mmol L^−1^ NaCl (scan rate 100 mV s^−1^); (**b**) surface conductivity vs. thickness of gold layer; (**c**) surface conductivity vs. literature values of CNT membranes; (**d**) literature data of conductive membranes.

Cyclic voltammetry experiments (Figure 4a) show that with increasing ionic strengths and electric conductivity of the feed solution, the current increased as well. However, no specific peaks can be observed, which leads to the conclusion that electrochemical reactions are negligible during the filtration experiment [23]. Furthermore, experiments showed that the distribution of the applied potential to cathode and anode was almost equally distributed. When applying 2 V of cell voltage with the potentiostat, anode surface potential was measured at +1 V vs. Ag/AgCl and cathode surface potential was −1 V vs. Ag/AgCl. For a membrane surface potential of ±1 V vs. Ag/AgCl, a current of around ±1 mA is measured for a surface area of 42 cm^2^, leading to a current density of 0.024 mA cm^−2^, which can be considered as too low for significant electrochemical activity [7]. The experiments also showed that the membrane coating remains stable up to relatively high [23] catholic and anodic potentials up to ± 4 V vs. Ag/AgCl. 

Investigation of surface conductivity after sputter coating of the membrane shows that the layer thickness (5, 10 and 15 nm Au) has a major impact on the resulting conductivities (Figure 4b). This can be explained by the proceeding coverage of the virgin surface with gold. Other studies claim that, depending on the surface roughness and morphology, at least 10 nm of gold is needed to generate a homogeneous and completed gold surface overcoming occurring isolated Au-clusters [12,24].

Through utilization of a sputtered gold layer of 15 nm, a relatively high surface conductivity is generated. Compared to the recently developed carbon nanotube (CNT) membranes [7,23,25,26], the conductivity is at least three orders of magnitudes higher (Figure 4c,d). 

Taking into account the small currents flowing during electro-repulsive membrane filtration experiments [27], and considering Ohm´s law, it is probably not necessary to have moderately high surface conductivities to see an effect on fouling and rejection behavior. This is also proved by the results of several mentioned publications, which focus on fouling mitigation. Though, by using the membrane for electro-oxidation [28,29,30] or electro-reduction [31] reactions, the conductivity does have a major influence on the filtration performance [9]. 

### 3.2. Filtration Experiments

#### 3.2.1. Effects of Cathodic and Anodic Potentials

**Figure 5:**
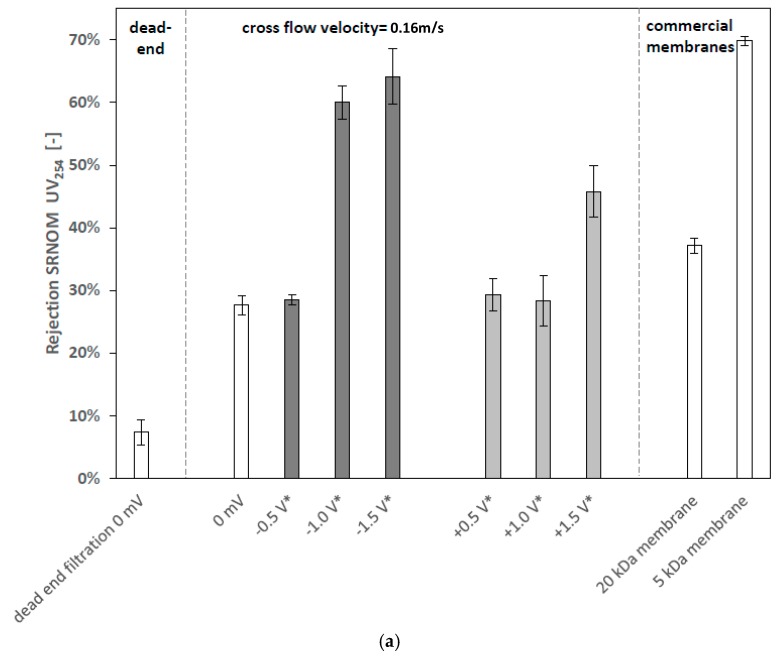

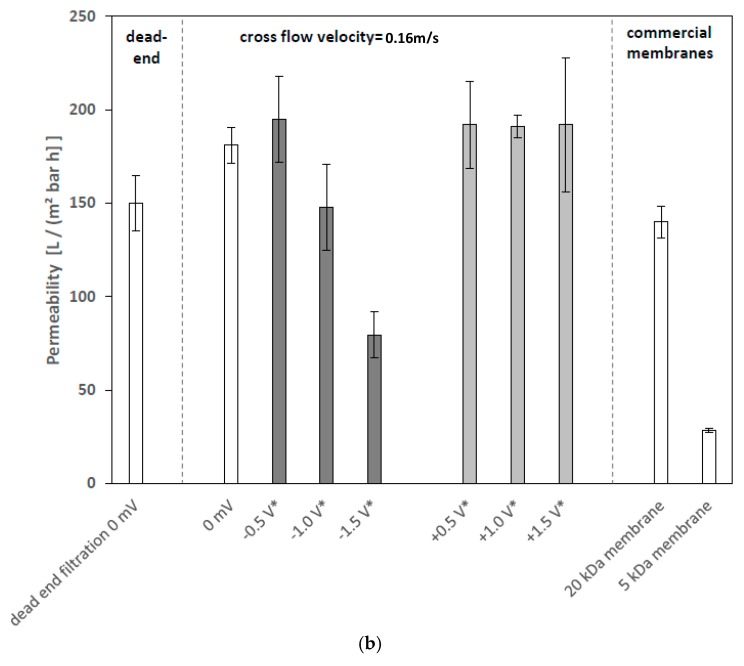
UV_254_ rejection rates and permeability data; all experiments are conducted at least in triplicates with membrane surface of 42 cm^2^, transmembrane pressure (TMP) of 1 bar, cross-flow velocity of 0.16 m s^−1^ (except of dead-end experiment), sample for rejection calculation is taken after filtration of 250 mL, feed: 6 mg L^−1^ of SRNOM, 1 mmol L^−1^ NaCl, pH 7; (**a**) rejection data after 250 mL of filtration; (**b**) permeability data after 250 mL of filtration, * all potentials are measured vs. Ag/AgCl reference electrode, error bars present standard deviations.

Dead-end filtration of the SRNOM feed solution without applied potential confirm that there is only 6% UV_254_ rejection (Figure 5a), which is based on steric exclusion effects [3,32]. Rejection of 28% can be measured during cross-flow filtration without applied potential. This leads to the conclusion that the increased rejection is mostly based on electrostatic repulsion, which is the rejection mechanism that is mainly affected by concentration polarization [3,5].

No significant change in rejection was observed by applying −0.5 V vs. Ag/AgCl. However, when raising surface potential to −1 V vs. Ag/AgCl the UV_254_ rejection abruptly increases from 28% to 60%. Obviously, the application of negative potential to membrane surface leads to the enhancement of rejection with increasing potential, though the rejection enhancement is not proportional to the applied potential. A similar behavior was also observed by other authors [7,23,33] and might be explained by the concept of counteracting drag force and electrostatic repulsion force which results in a critical electric field or respective applied surface potential [23,33]. Through further increase of voltage to −1.5 V vs. Ag/AgCl, UV_254_ rejection reached up to 64%. Corresponding permeability of the negatively charged membranes shows that with increasing negative potential and respective increasing rejection, permeability decreases (Figure 5b). This is probably connected with a growing influence of concentration polarization [34]. This assumption is supported by the observation of a sudden increase of flux and decrease of rejection after changing surface potential from −1 V to +1 V vs. Ag/AgCl during the experiment (see Section 3.32.3).

Comparing the UV_254_ rejection rate of commercial 20 and 5 kDa membranes to the externally charged 150 kDa membrane, it can be stated that due to the applied potential of −1.5 V vs. Ag/AgCl the 150 kDa membrane enhances rejection ability into the range of a 5 kDa membrane. Throughout, the permeability of the externally charged UP150 is still more than three times higher than that of the 5 kDa membrane.

When applying +0.5 and +1 V vs. Ag/AgCl, no observable effects on UV rejection rate can be observed; permeability is also unchanged in relation to the uncharged membrane. With application of +1.5 V vs. Ag/AgCl, UV_245_ rejection increases to 45% while corresponding permeability does not decrease (Figure 5b). Rejection enhancement at +1.5 V vs. Ag/AgCl might be explained by electro-sorption of negatively charged NOMs onto the positive membrane surface. 

Dudchenko et al. [23] also observed strong fouling enhancement of an electro-conductive UF membrane through filtration of sodium alginate solution when positive potentials were applied. This can be interpreted as an electro-sorption onto the membrane surface as well, with the difference that relatively large alginate molecules were not able to pass membrane pores as NOM molecules can do. 

#### 3.2.2. Influence of Ionic Strengths and pH

**Figure 6:**
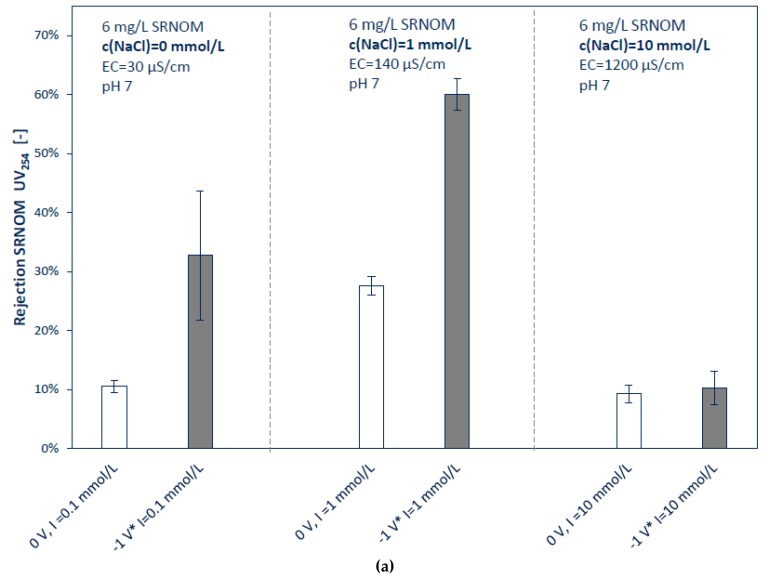

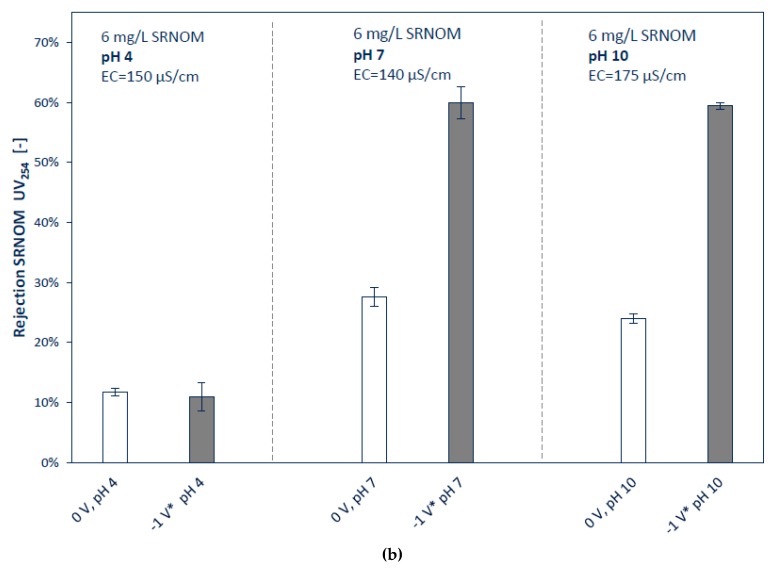
Influence of ionic strengths (**a**) and pH (**b**) on rejection; all experiments are conducted at least as triplicate with a membrane surface of 42 cm^2^, TMP of 0.1 MPa, cross-flow velocity of 0.16 m s^−1^, sample for rejection calculation is taken after filtration of 250 mL; feed: 6 mg L^−1^ of SRNOM, pH 7, NaCl concentration of (**a**) 0, 1, 10 mmol L^−1^, (**b**) pH 4, 7 and 10; * all potentials are measured vs. Ag/AgCl reference electrode, error bars present standard deviations.

As expected, the ionic electrolyte background shows to have a major influence on rejection behavior (Figure 6a). Other authors revealed that ultrafiltration membranes show less rejection of NOMs with increasing ionic strengths [1,35]. It is considered that with raising ionic strengths the shielding of the organic molecules is also increasing, which leads to a lesser effective surface charge. The same applies to the membranes surface itself [1]. At high charge and low ionic strengths, the NOM molecules are more linear stretched because of repulsive functional groups (carboxylic groups), and at low charge and higher pH the NOM molecules curl up and have a more compact structure [36].

However, experiments indicate that the concentration of 1 mmol L^−1^ NaCl, compared to no ionic background, led to an increase of rejection of the externally charged and uncharged membrane. This might be explained by the higher electrical double-layer capacitance induced by the rise of the ionic strengths [7]. With almost no ions in the feed solution, no double layer can be formed. This can also be seen by the results of cyclic voltammetry. At low ionic concentrations, flowing current is much lesser. Shao et al. [5] observed the same results by filtration of humic acids (at ionic strengths of 0 and 3 mmol L^−1^) with neutral and negatively charged UF membranes. Specific adsorption of ions might be a reason for increased rejection also [37].

At ionic strengths of 10 mmol L^−1^, UV_254_ rejection rate decreases to 10%. At this ion concentration, the repulsion of electrostatic field might collapse and the remaining rejection bases mainly on steric exclusion. At this ionic strength, external charging of the membrane cannot enhance the rejection rate any further.

At pH 4, rejection is only about 10%, which can be considered the fraction, which is rejected by steric exclusion (Figure 6b). Below pH 4.7, SRNOM is neutrally charged [38], therefore no electrostatic repulsion is present. Furthermore, the pH at point of zero charge (pH_pzc_) of gold is 4.9 [39]. In this regard, it is plausible that the application of negative potential of −1 V vs. Ag/AgCl does not result in further impact on rejection. At pH 7, rejection increases to 29% for the not externally charged membrane. This might be explained by the negative zeta potential that naturally develops at the membrane’s gold surface even without connected potentiostat [22]. At pH 7, deprotonation of carboxylic groups of the NOMs are almost completed. However, negative charge density of NOMs is still increasing by raising the pH to 10 [40]. Nevertheless, no additional rejection can be observed at pH 10 compared to pH 7.

#### 3.2.3. Changing Sign of Surface Potential during Filtration

**Figure 7:**
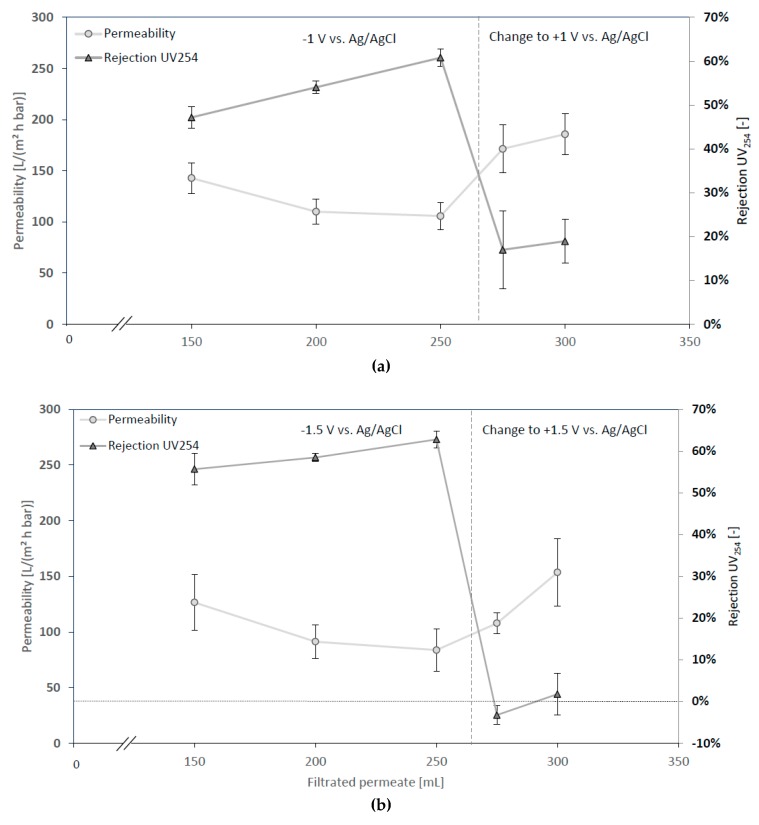
Filtration experiment with changing surface potentials; feed: 6 mg L^−1^ of SRNOM, 1 mmol L^−1^ NaCl, pH 7; (**a**) applied surface potential −1 V changed to +1 V vs. Ag/AgCl after 250 mL filtration; (**b**) applied surface potential −1.5 V changed to +1.5 V vs. Ag/AgCl after 250 mL filtration; error bars present standard deviations.

For further investigation on the impact of surface charge, the potential was changed during running filtration experiments. Immediately after the change of signs from negative to positive surface charge, the rejection decreases and the flux increases (Figure 7a). The increase of flux might be explained by the decay of previously developed concentration polarization [34]. The effect of increasing flux is even more pronounced when the applied potential was more elevated (±1.5 instead of ±1 V) (Figure 7b). After changing potential from −1.5 to +1.5 V vs. Ag/AgCl, a negative rejection of −5% was measurable for a short time, which supports the hypothesis of an electrostatically induced concentration polarization, which decays away by the change of signs. Another explanation for negative UV_254_ rejection rates might be the sudden desorption of NOMs from the membrane’s surface. However, this explanation seems unlikely as the surface was negatively charged like the NOMs, which does not facilitate adsorption.

## 4. Discussion and Conclusions

### 4.1. Membrane Modification with Gold Sputter Coating

Sputter coating of UF membranes with ultrathin gold layers is a simple and suitable method to transform a commercial flat sheet membrane into an electro-conductive UF membrane. A 40% decrease in permeability results under the present conditions; nevertheless, other parameters as MWCO or hydrophobicity remained unchanged (see Section 3.1). SEM imaging confirms the development of a secondary gold layer on the membrane surface when 15 nm of gold was sputtered. Accordingly, resulting permeability decrease is presumably caused by higher filtration resistance due to the additional passage of water through the secondary layer of gold and not because of the reduction of the nominal pore size of the membrane [12].

The resulting surface conductivity after 15 nm gold coating is relatively high; compared with CNT membranes, it is three orders of magnitudes higher (see Figure 4c). Accordingly, Au-coating of only 5 nm gold on the present PES-UF membrane is enough to obtain sufficient surface conductivities. Another advantage of Au-coating is its chemical stability. The membranes can be used as anode and cathode which is not possible with widely investigated CNT membranes [25,26]. These membranes are only stable to cell potential of 1.5 V when the CNT membrane is used as an anode [23]. No decrease of surface conductivity was evident after filtration experiments even if the membrane was used for many times in a row; furthermore, no gold particles or deposits could be found at any time in the feed tank. This leads to the assumption that the coated gold layer is also mechanically stable and not affected by the shear stress of the applied cross-flow.

It might be interesting to utilize the present effects even on a commercial basis. Au-coating of polymer microfiltration, UF, and even nanofiltration membranes seems feasible. The additional material cost for gold coating of flat sheet membranes for a 15 nm coating sums up to around 8 US $/m^2^. Conceivably, it is possible to use titanium or silver instead of gold for more economic fabrication. By sputtering the counter electrode on the support layer of the membrane and the working electrode on the active layer, one urgent drawback of the implementation of electrical conductive membranes in spiral wound modules could be overcome [27], because no additional counter electrode is necessary. However, further studies on material stability, overall fouling, rejection and real water matrix behavior are necessary.

### 4.2. Filtration Experiments with Applied Surface Potential

The zeta potential has become the most important measurable reference for evaluating the surface charge of membranes [16,41]. An externally induced zeta potential is difficult to measure and complex to model [39,42,43]. It is influenced by several parameters as applied potential to the electrode, pH, ionic strength, electrode distance and electrode material [5,44]. Duval et al. [22] modeled the double layer potential of a gold electrode which can be assumed to be comparable to the zeta potential with respect to applied potential, pH and ionic strengths [45]. Barten et al. [39] confirmed the results of the model with an experimental approach. Both found a strong dependence of ionic strengths and pH on the induced zeta potential. At ionic strengths of approx. 1 mmol L^−1^ the induced zeta potential diminishes to 1/10 of the applied electrode potential vs. Ag/AgCl. Accordingly, the application of −1000 mV vs. Ag/AgCl resulted in a zeta potential of −99 mV for I = 1 mmol L^−1^, −65 mV for I = 10 mmol L^−1^ and −35 mV for I = 100 mmol L^−1^. 

The present results of filtration experiments on charged membranes confirm the earlier proposed electrostatic rejection mechanism [3,4,5,37]. In interplay with pore size, external negative surface charge led to an increase of NOM rejection starting at the potential of −1 V vs. Ag/AgCl (see Section 3.2.1). After lowering the molecule charge of the applied NOMs by the decreasing of pH or increasing of ionic strengths, NOM rejection enhancement effects induced by the externally applied potential was not pronounced anymore (see Section 3.2.2). Considering these, arguments that measured rejection rates are caused by chemical oxidation/reduction or the building gas bubbles (e.g. oxygen or hydrogen) on membrane surface are less convincing. In this case, rejection enhancement shall also be visible when NOMs charge is diminished. The external charging of the membrane probably still has an effect on the resulting zeta potential of the membrane at different ionic strengths [39], but because the NOMs are less charged, no electrostatic repulsion is induced.

Due to external charging, filtration performance can be improved considerably. The application of −1.5 V vs. Ag/AgCl on the Au-coated UF membrane with 150 kDa MWCO results in rejection performance close to a 5 kDa membrane at much higher permeability. Throughout, additional energy consumption due to membrane charging is approx. 0.001–0.01 kWh per cubic meter filtrated water, which is negligible compared to necessary energy for pumping.

**Figure 8:**
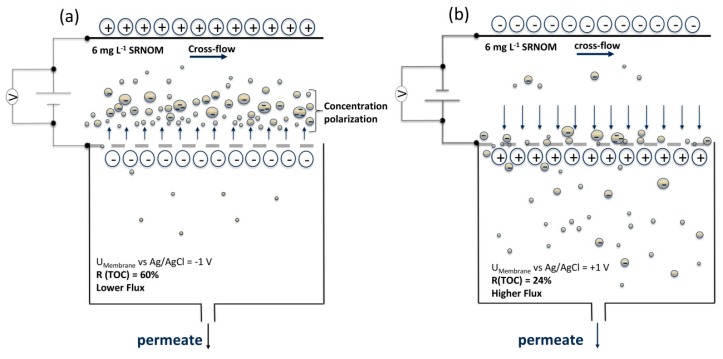
Model of NOM rejection on membrane surface with anodic and cathodic potential; (**a**) negatively charged membrane with high rejection due to electrostatic repulsion resulting in strong concentration polarization; (**b**) positively charged membrane with no electrostatic repulsion, higher flux and less rejection.

The change of sign of applied potential during the filtration experiment reveals that NOM rejection rate drops to zero after reversing potentials. Simultaneously, the corresponding flux is increasing (see Section 3.2.3). This results support the assumption that NOM rejection was caused mainly by electrostatic mechanisms. In Figure 8, a graphical model of NOM rejection based on the described observations is presented.

In conclusion, results show that in-situ charging of the investigated electro-conductive Au-membrane results in a possible functionalization of the material by tunable membrane charge. By this, a promising and in particular flexible tool is available to further evaluation of the impact of surface charge in UF membrane filtration with respect to the manifold separation tasks. This is part of ongoing research.

## Figures and Tables

**Table 1:** Parameters of filtration experiments.

Tested Parameter	Membrane Surface Potential	pH of Feed	Ionic Strength Feed
Membrane Surface Potential	**−500, −1000, −1500, +500, +1000, +1500, 0 mV vs. Ag/AgCl**	7	1 mmol L^−1^
pH of Feed	0, −1000 mV	**4, 7, 10**	1 mmol L^−1^
Ionic Strength Feed	0, −1000 mV	7	**0, 1, 10 mmol L^−1^**

## Supplementary Materials

The following are available online at http://www.mdpi.com/2077-0375/8/3/64/s1. Figure S1: Contact Angle Measurement: (a) experimental set-up: A) camera B) Stand C) Box D, E, F) Sample holder G) lamps; (b) software aided analyses of contact angle (Foto: L. Gormsen), Figure S2: Electrical surface conductivity: Van-der-Pauw-Method (Nolte 2009) (a) scheme for contacting membrane (b) nomogram for determining correction factor (Van der Pauw 1958), Figure S3: Influence of ionic strengths and pH on rejection and corresponding permeability: all experiments are conducted at least as triplicate with membrane surface of 42 cm^2^, TMP of 0.1 MPa, cross-flow velocity of 0.16 m s^−1^, sample for rejection calculation is taken after filtration of 250 mL; feed: c(SRNOM) = 6 mg L^−1^, pH 7, c(NaCl) = 0, 1, 10 mmol L^−1^ (b) pH 4, 7 and 10; *all potentials are measured vs. Ag/AgCl reference electrode, error bars present standard deviations.
