# Supplementary material for: Electro-Conductive Composite Gold-Polyethersulfone-Ultrafiltration-Membrane: Characterization of Membrane and Natural Organic Matter (NOM) Filtration Performance at Different In-Situ Applied Surface Potentials
Source: Membranes (Basel). 2018 Aug 16;8(3):64. doi: 10.3390/membranes8030064 (PMC6160915; doi:10.3390/membranes8030064)
Supplement: Supplementary file 1 [file membranes-08-00064-s001.pdf]

Supplementary Materials

1. Membrane Characterization

1.1. Hydrophobicity—Contact Angle

Experimental set-up of contact angle measerment is presented in Fig. 1.

(a) (b)

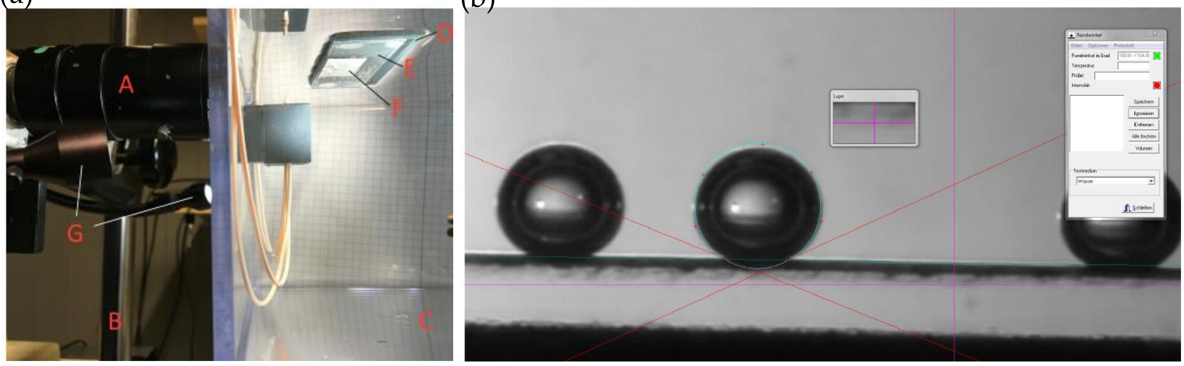

Figure 1. Contact Angle Measurement: (a) experimental set-up: A) camera B) Stand C) Box D, E, F) Sample holder G) lamps; (b) software aided analyses of contact angle (Foto: L. Gormsen).

1.2. Electrical Properties of Conductive Membrane Electrode

(a) (b)

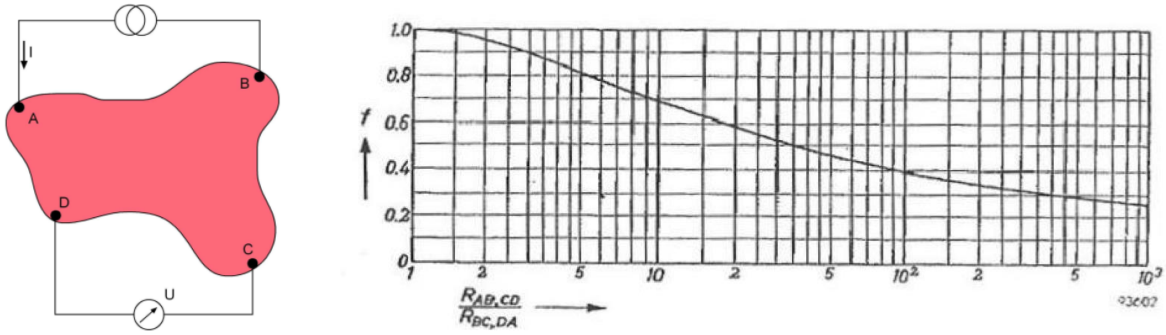

Figure 2. Electrical surface conductivity: Van-der-Pauw-Method (Nolte 2009) (a) scheme for contacting membrane (b) nomogram for determining correction factor (Van der Pauw 19.58)

A membrane sample is contacted as in Fig. 2 at point A, B, C and D. A current I_{AD} is flowing from A to D and the voltage drop U_{DC} is measured from D to C. After Ohm´s Law $R_{AB,CD}$ is calculated. Through turning of the sample with an angle of 90° current I_{BC} and U_{DA} is determined. Moreover, correction factor f is calculated. Surface conductivity is determined following equation 1. The reciprocate of ρ is the surface conductivity.

$$\rho = \frac{\pi \cdot d}{2 \ln 2} \frac{R_{AB,CD} + R_{BC,DA}}{2} \cdot f\left(\frac{R_{AB,CD}}{R_{BC,DA}}\right) \quad (1)$$

2. Filtration Experiments

In Fig. 3 additional permeability data is presented for filtration experiments with varying pH and ionic strengths.

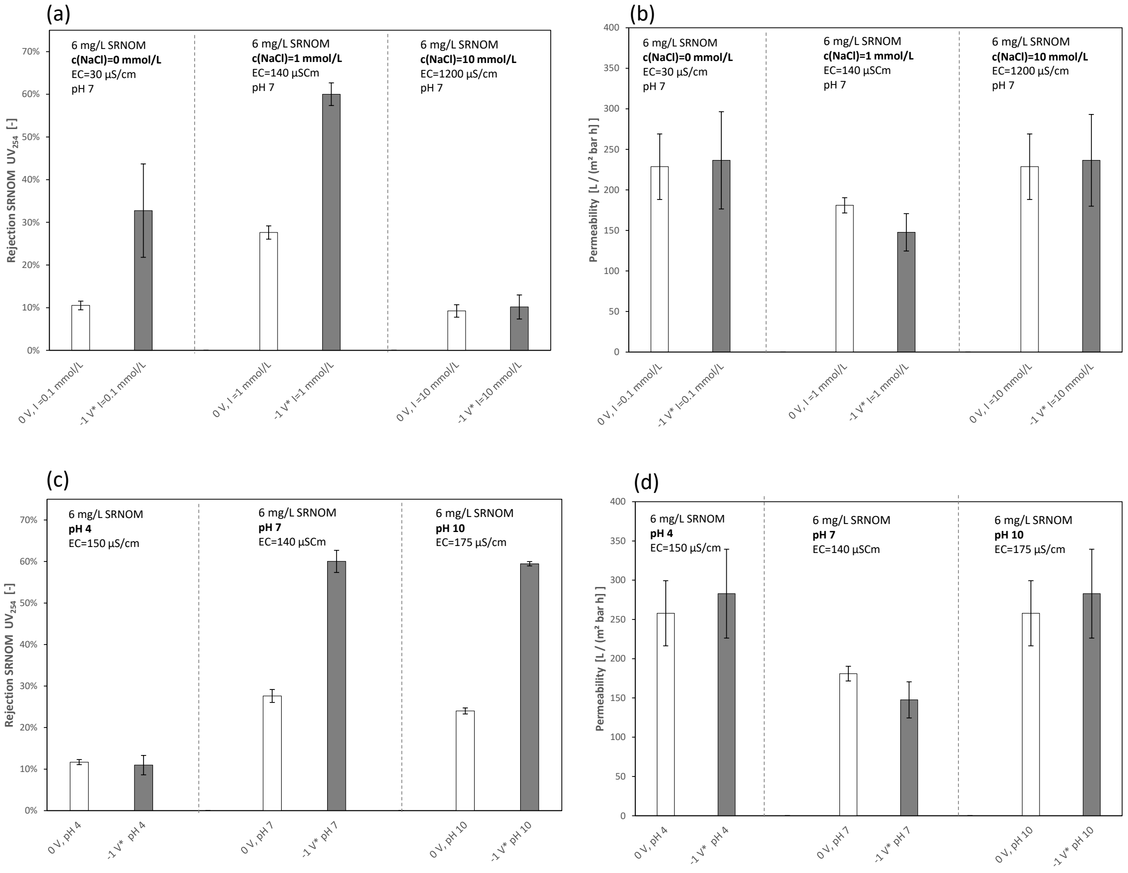

Figure 3. Influence of ionic strengths and pH on rejection and corresponding permeability: all experiments are conducted at least as triplicate with membrane surface of 42 cm², TMP of 0.1 MPa, cross-flow velocity of 0.16 m s^{-1}, sample for rejection calculation is taken after filtration of 250 mL; feed: c(SRNOM) = 6 mg L^{-1}, pH 7, c(NaCl) = 0,1, 10 mmol L^{-1} (b) pH 4, 7 and 10; *all potentials are measured vs. Ag/AgCl reference electrode, error bars present standard deviations.

References

Nolte, Mathias C. M. (2009): Elektrisch leitfähige Umkehrosmosemembranen zur Verminderung des Biofoulings. Dissertation. Hamburg: Selbstverlag.

Van der Pauw, J. (1958): A method of measuring the resistivity and Hall coefficient on lamellae of arbitrary shape. In: *Philips Technical Review* (20), S. 220–224.
